# The role of acid sensing ion channels in the cardiovascular function

**DOI:** 10.3389/fphys.2023.1194948

**Published:** 2023-06-14

**Authors:** Omar López-Ramírez, Antonia González-Garrido

**Affiliations:** ^1^ Instituto de Oftalmología Fundación de Asistencia Privada Conde de Valenciana, I.A.P., Mexico City, Mexico; ^2^ Laboratorio de Enfermedades Mendelianas, Instituto Nacional de Medicina Genómica, Mexico City, Mexico

**Keywords:** baroreceptor, ischemic pain, chemoreceptor, myocardium acidosis, ASIC cardiovascular

## Abstract

Acid Sensing Ion Channels (ASIC) are proton sensors involved in several physiological and pathophysiological functions including synaptic plasticity, sensory systems and nociception. ASIC channels have been ubiquitously localized in neurons and play a role in their excitability. Information about ASIC channels in cardiomyocyte function is limited. Evidence indicates that ASIC subunits are expressed in both, plasma membrane and intracellular compartments of mammalian cardiomyocytes, suggesting unrevealing functions in the cardiomyocyte physiology. ASIC channels are expressed in neurons of the peripheral nervous system including the nodose and dorsal root ganglia (DRG), both innervating the heart, where they play a dual role as mechanosensors and chemosensors. In baroreceptor neurons from nodose ganglia, mechanosensation is directly associated with ASIC2a channels for detection of changes in arterial pressure. ASIC channels expressed in DRG neurons have several roles in the cardiovascular function. First, ASIC2a/3 channel has been proposed as the molecular sensor of cardiac ischemic pain for its pH range activation, kinetics and the sustained current. Second, ASIC1a seems to have a critical role in ischemia-induced injury. And third, ASIC1a, 2 and 3 are part of the metabolic component of the exercise pressure reflex (EPR). This review consists of a summary of several reports about the role of ASIC channels in the cardiovascular system and its innervation.

## Graphical Abstract

**Figure:**
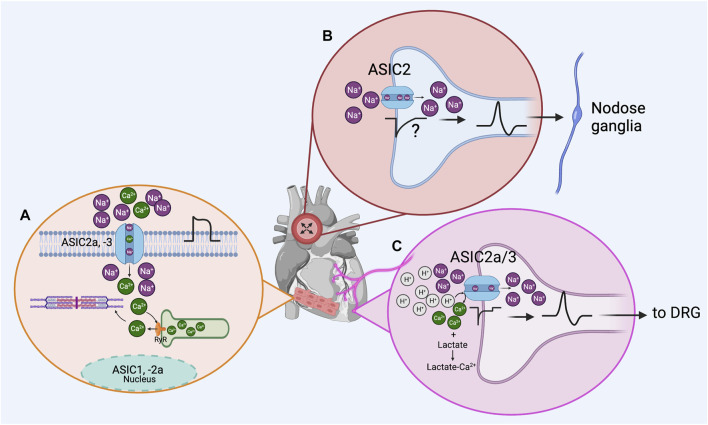
Acid Sensing Ion Channels (ASIC) in the cardiovascular system. **(A)**, according to experimental findings, ASIC1, -2a and −3 subunits are expressed in myocardium. ASIC2a and −3 could be forming functional channels permeating Na^+^ and Ca^2+^ ions, depolarizing the membrane to facilitate action potential firing, and contributing to Ca^2+^ dependent contraction. Localization of ASIC1 and 2a subunits in the nucleus of cardiomyocytes suggests an unrevealed role of ASIC channels in the myocardium physiology. **(B)**, in the baroreceptor reflex, ASIC2-containing channels present in nodose ganglion terminals innervating the aorta arch act as mechanosensors and are part of the baroreceptor activated by aorta stretching. **(C)**, ASIC channels act as chemotransducers in the dorsal root ganglia (DRG) innervating the heart; activation of ASIC3-containing channels by protons and enhanced by lactic acid release in DRG neurons innervating the heart contributes to ischemic pain sensation (Created with BioRender.com).

## 1 Introduction

ASICs are proton-gated cation channels members of the amiloride-sensitive degenerin/epithelial Na channel (DEG/ENaC) superfamily. Six different subunits encoded by four genes have been cloned in neuronal tissue: ASIC1a, ASIC1b, ASIC2a, ASIC2b, ASIC3 and ASIC4 (Waldmann et al., 1997; Gründer et al., 2000; Benson et al., 2002; Bianchi & Driscoll, 2002). Each ASIC subunit comprises two transmembrane domains connected by a long extracellular loop rich in cysteine residues, with the amino and carboxyl termini located intracellularly (Saugstad et al., 2004). Structure of ASIC1a homomeric channels was determined by X-ray crystallography, showing that ASICs assemble as trimers (Jasti et al., 2007). Functional channels can be trimers from the same subunit (homomers) or the combination of different subunits (heteromers) (Hesselager et al., 2004). ASIC2b and ASIC4 subunits do not form functional homomeric channels (Lingueglia, 1997; Akopian et al., 2000; Gründer et al., 2000).

ASIC channels are sensors of extracellular protons in a pH range from 7 to 4.0. ASIC kinetics depend on the subunits conforming the channel. Thus, the ASIC1a, -1b and −3 homomeric channels display fast desensitization kinetics, whereas the ASIC2a homomeric channel has slow desensitization kinetics (Diochot et al., 2007). The affinity for protons varies for each subunit, ASIC3 is the most sensitive (pH_50_ of 6.4) and ASIC2 the least sensitive (pH_50_ of 4.5). For ASIC1 variants the pH_50_ is similar, 5.8 and 6.1 for ASIC1a and ASIC1b, respectively (Hesselager et al., 2004).

ASICs channels are highly selective for Na^+^ and have low permeability to other monovalent inorganic cations. ASIC1a subunit is also permeable to Ca^2+^ ions (PNa^+^/PCa^2+^ of 2.5) (Waldmann et al., 1997; Bässler et al., 2001). This fact makes this subunit particularly relevant since Ca^2+^ regulates many physiological functions including neurotransmitter release and muscle contraction, and several other as an intracellular second messenger. Moreover, increased Ca^2+^ influx can also lead to increased cell death secondary to acidosis.

## 2 ASIC channels expressed in the myocardium

The cardiac muscle or myocardium is responsible for the mechanical (pump) functioning of the heart. Proper cardiac functioning depends on synchronized excitation (electrical) and contraction (mechanical) in highly specialized regions of the heart, including sinoatrial node, atria, atrioventricular node, His-Purkinje system, and ventricles. Specific electrical properties arise from the variety of ion channel expression in each region.

In neurons, ASIC channels are known to modulate their electrical activity and thus their function.

There are very limited reports of expression of ASIC channels in the adult mammalian myocardium and some of their results are contradictory. ASIC1, 2a and 3 are expressed in the adult rat myocardium with different distribution. ASIC1 is mostly in the nucleus, ASIC2a is uniformly distributed in the cytoplasm and nucleus and ASIC3 is in the cytoplasm. Since ASIC2a and 3 are localized at subcellular level, they might also play intracellular functions in mammalian myocardium (Hu et al., 2017). ASIC channels in the myocardium are most likely contributing to its electrical activity, since acidic extracellular pH elicits ASIC-like inward currents in neonatal and adult rat cardiomyocytes, that are partially blocked by amiloride and PcTx1 (Hu et al., 2017). In addition, ASIC-like currents were also observed in human induced pluripotent stem cell-derived cardiomyocytes (hiPSC-CMs), derived from three different hiPSC lines (IMR-90, iPSC-K3, and Ukki011-A). However, in this same study, ASIC-like currents and their protein subunits were absent in adult rat cardiomyocytes (Zhang et al., 2019). This evidence indicates the functional expression of ASIC channels in both, the developing and adult mammalian myocardium.

Analysis of mRNA sequencing and ribosome profiling data collected from human left ventricular cardiac tissue revealed the expression of ASIC1 and 3 subunits (Redd et al., 2021). Furthermore, genome-wide association analysis revealed a significant association between human polymorphisms in ASIC1a and heart ischemic diseases (coronary heart disease and myocardial infarction) (Redd et al., 2021). Besides, according to AceView database (developed at National Center for Biotechnology Information, NCBI), ASIC 1, 3 and 4 but not ASIC 2 transcripts are expressed in the human heart. Being ASIC3 transcript the most abundant. The repository was accessed at https://www.ncbi.nlm.nih.gov/IEB/Research/Acembly/index.html?human; and the name of genes consulted were: ACCN2 for ASIC1, ACCN1 for ASIC2, ACCN3 for ASIC3 and, ACCN4 for ASIC4.

## 3 ASIC channels in the cardiac afferent innervation

The heart has a dual afferent innervation mediated by the vagal and sympathetic nerves. The bodies of vagal afferents are in the nodose ganglia, and their central projections reach the brainstem. While sympathetic neuronal bodies reside in the lower cervical and upper thoracic of dorsal root ganglion (DRG) and their axons follow the sympathetic nerve to innervate the epicardium (Malliani et al., 1986; Foreman, 1999). ASIC channels in cardiac afferent terminals are thought to play a dual role, in sensory terminals from the nodose ganglia, they detect changes in arterial pressure (mechanosensitive) and, in terminals from the DRG, they are activated by changes in pH (chemosensitive) which are processed as pain chest or angina.

### 3.1 ASIC in baroreceptors

The arterial baroreceptor reflex regulates acute fluctuations of arterial blood pressure (ABP) during changes in exercise, posture, stress and other conditions. In response to these fluctuations the brainstem regulates the parasympathetic and sympathetic systems activities to compensate them. An increase in blood pressure, causes an increase in the parasympathetic activity and a decrease the sympathetic activity. And *vice versa*, a decrease in blood pressure elicits a decrease in the parasympathetic activity and an increase in the sympathetic nerve activity (Benarroch, 2008).

The arterial baroreceptors are mechanosensitive afferent nerve terminals located in the adventitia of the carotid sinuses (innervated by the glossopharyngeal nerve) and aortic arch (innervated by the vagus nerve). Mechanotransduction in peripheral baroreceptor afferents involves DEG/ENaC and ASIC. ASIC subunits are expressed in neuron populations from DRG, trigeminal and nodose ganglia innervating the arterial baroreceptor nerve endings located in the aortic arch and carotid sinus (Drummond et al., 1998; Molliver et al., 2005). ASIC2b transcript has predominance over ASIC1a, 1b, 2a, and 3 in nodose ganglia and aortic baroreceptor terminals and ASIC2 protein colocalized with ASIC1 and ASIC3 in some but not all neurons, suggesting the presence of heteromeric channels (Lu et al., 2006). ASIC2 null mice presented reduced spontaneous baroreflex sensitivity causing severe dysregulation of neurohumoral control of the circulation, reproducing the dysautonomia seen in human hypertension and heart failure, and impaired baroreceptor mechanosensitivity (Drummond et al., 1998; Lu et al., 2006; Lu et al., 2009). This evidence suggests that ASIC2 is essential for the proper mechanosensation of baroreceptor neurons, and it is highly likely that ASIC2 is part of the pressure-sensing channel of the baroreceptor.

Evidence of ASIC channels, particularly ASIC2 subunits required in mechanosensory processes has been also found in peripheral cutaneous and gastrointestinal nerves (Price et al., 2000; Page et al., 2005). Nevertheless, the mechanism of ASIC channel in mechanosensation is not yet clear. It has been proposed a homologous mechanism of the tether model described in *Caenorhabditis elegans*, in which the extracellular matrix and the cytoskeleton proteins act like gating-spring to mechanically activate ASIC channels (Chalfie, 2009). In mammals, stomatin-domain proteins, PDZ-domain proteins and RGD (arginylglycylaspartic acid) ligands binding to integrins (collagen, laminin, fibronectin) are proposed to act as tethers to ASIC channels in skin mechanoreceptors (Cheng et al., 2018; Ruan et al., 2021).

### 3.2 ASIC and myocardial ischemia

Ischemic heart disease is the leading cause of mortality worldwide (WHO PAGE https://www.who.int/news-room/fact-sheets/detail/the-top-10-causes-of-death). Heart ischemia is the response to reduced blood flow and the subsequent lack of oxygen and nutrients to tissues and a declination in the contractile function. The lack of oxygen in the myocardium compromised aerobic cellular metabolism resulting in ATP reduction. Depletion of ATP causes failure of the Na^+^/K^+^ pump producing cellular swelling as Na^+^ and water are held within the cell. Increasing Na^+^ intracellular concentration results in an overall plasma membrane depolarization and Ca^2+^ influx, which overexcites the cell causing formation of reactive oxidative species (ROS). ROS damage the cell producing an overall extracellular acidosis. If ischemia episode is prolonged, anaerobic metabolic pathways prevail resulting in additional extracellular acidosis by lactic acid synthesis (Romanelli et al., 2020). In the ischemic area the pH can drop down to 6.0–6.8 (Gabel et al., 1997; Gorodetsky et al., 2016), more than sufficient to activate ASIC channels. Sustained ischemia leads to myocardial infarction, which results in necrosis of a significant portion of the myocardium (Yellon & Hausenloy, 2007). Extracellular acidosis is detected in neuronal terminals innervating the myocardium by several chemosensitive receptors including ASIC channels resulting in the sensation of pain and activation of autonomic reflexes.

Cardiac sensory terminals from DRG express ASIC subunits in high levels, they have large inward currents activated by acidic pH and fire action potentials in response to extracellular acidification (Garza et al., 2010; Garateix et al., 2011; Mercado et al., 2015). Since in the myocardial ischemia there is a drop in the extracellular pH and overexcitation of the afferent innervation, ASIC channels have been postulated as one of the main candidates to be the molecular sensor of cardiac ischemic pain (Benson et al., 1999; Immke & McCleskey, 2001a; 2001b; Sutherland et al., 2001).

Experimental data indicate that native acid receptors in the cardiac sensory DRG neurons share several features with ASIC2a/3 channels (Benson et al., 1999; Sutherland et al., 2001; Yagi et al., 2006; Hattori et al., 2009). ASIC2a/3 channels have faster gating kinetics between closed, open, and desensitized states than other ASIC channels, consistent with the binding of multiple protons (Sutherland et al., 2001). ASIC2a/3 channels rapidly desensitize but have a sustained current that persist to prolonged acidosis, over tens of minutes (Yagi et al., 2006).

Paradoxically, restoring the coronary blood flow after an ischemic episode can induce the death of cardiomyocytes. This is known as ischemia-reperfusion injury (IRI). If the ischemic episode is prolonged, the IRI adds damage to the infarct zone. Myocardial infarction and IRI are significant postoperative complications that limit survival after heart surgery. During IRI many cellular changes occurs including ionic changes, such as intracellular calcium accumulation and, intra and extracellular protons accumulation. The resulting acidosis activates several membrane ion channels and receptors, including the ASIC channels. Particularly, ASIC1a currents are potentiated during ischemia by the increased levels of extracellular lactate (Immke & McCleskey, 2001a; Immke & McCleskey, 2001b; Immke & McCleskey, 2003). ASIC1a mediates ischemia-induced neuronal death, and its inhibition has neuroprotective effects on the brain and the retina (Xiong et al., 2004; Chassagnon et al., 2017; Dibas et al., 2018; Qiang et al., 2018). In the heart, inhibiting ASIC1a by specific blockers or genetic ablation attenuates IRI and improves the recovery of heart function (Redd et al., 2021). This is enough evidence as proof of concept for the inhibition of ASIC1a to protect excitable cells from death after ischemia episodes.

On the other hand, ASIC3 role in heart ischemia has the opposite effect as ASIC1a. The expression of ASIC3 plays a critical role in mediating protection against heart ischemia-induced fibrosis in a murine ischemia model (Cheng et al., 2011). In addition to initiate autonomic reflexes, activation of ASIC3 elicits neuropetides release such as calcitonin gene-related peptide, substance P and neurokinins, which have been proposed to play a protective role against ischemia and reperfusion injury (Ustinova et al., 1995; Bolli & Abdel-Latif, 2005).

Finally, the study of the participation of ASIC channels in cardiac ischemic pain has led to the discovery of novel mechanisms of their gaiting. Lactate, produced during an ischemic event, is one of substances proposed to trigger cardiac ischemic pain. Experimental findings revealed that lactate enhances ASIC affinity to protons because of its capability to chelate extracellular divalent ions such as Ca^2+^, Mg^2+^ and Zn^2+^ which seems to be constitutively blocking ASIC channels. These results showed that ASIC channels opening requires not just proton binding but divalent cation unbinding (Immke & McCleskey, 2001a; Immke & McCleskey, 2001b; Immke & McCleskey, 2003).

### 3.3 ASIC and the exercise pressor reflex

The exercise pressor reflex (EPR), an autonomic feedback mechanism during exercise, causes a reduced parasympathetic tone and increased sympathetic activity, resulting in an augmented cardiac rate, contractibility, and blood pressure. EPR is the result of activation of thinly myelinated and unmyelinated group III and IV sensory endings from the DRG that innervate skeletal muscle (Mitchell et al., 1983; Adreani et al., 1997; Amann et al., 2014). It is thought that fibers from group III and IV respond mechanically (mechanosensitive) and chemically (metabosensitive), respectively, to skeletal muscle contraction.

Heart failure (HF) is a syndrome caused by impairment of ventricular filling or blood ejection resulting in a reduction in cardiac output and increased intracardiac pressures (Kurmani & Squire, 2017; McDonagh et al., 2021). It is one of the major causes of mortality and mobility worldwide (Tomasoni et al., 2019; Sinnenberg & Givertz, 2020; McDonagh et al., 2021). Patients with HF experience impaired tolerance for even mild exercise, exacerbated fatigue, and elevated cardiovascular risk most likely because they have an exacerbated EPR (Butenas et al., 2022).

ASIC1a, 2a and 3 subunits are highly expressed in afferent terminals innervating skeletal muscle (Gautam & Benson, 2013). Experimental data from animal models and healthy human testing suggest that ASIC channels contribute to the metabolic component by sensing the extracellular acidosis caused by muscle contraction of EPR (Hayes et al., 2007; Campos et al., 2019).

All three ASIC subunits expressed in afferent endings innervating skeletal muscle, 1a, 2a and 3, are mediating the signaling of the metabolic component of the EPR (Gibbons et al., 2015; Ducrocq et al., 2019; Li et al., 2023). Recently, ASIC1a was found to participate in the mechanical component of EPR in HF animals but not in healthy animals (Butenas et al., 2022). Taken together, this information suggests that ASIC-targeted strategies may have relevant clinical implications for patients with HF.

## 4 Concluding remarks

ASIC channels are widely distributed in excitable cells. There is evidence of ASIC subunits expressed in adult mammalian myocardium. Nonetheless, some evidence suggest that it could be an unrevealed role for ASIC channels at subcellular level. ASIC channels play a role during the development of cardiomyocytes. Innervation of the cardiovascular system has a dual role acting as acid sensors and as mechanosensors. Presence of ASIC2a subunits in aortic baroreceptor terminals has a central role in arterial pressure homeostasis. ASIC2a/3 channels detect acidosis during ischemic events translating it as pain sensation. ASIC1a inhibition is a promising therapeutic strategy to mitigate ischemia and reperfusion injury. ASIC1a, 2a and 3 contribute to the metabolic component of the EPR, and recent evidence suggest that ASIC1a play a role in the mechanical component as well. However, a more complete picture of the role of ASIC channels in the cardiovascular system is very much needed along with information of the mechanisms involved in the regulation of the cardiovascular function to explore to potential of ASIC-targeted therapies.
